# Myocardial T1 mapping and extracellular volume quantification: a Society for Cardiovascular Magnetic Resonance (SCMR) and CMR Working Group of the European Society of Cardiology consensus statement

**DOI:** 10.1186/1532-429X-15-92

**Published:** 2013-10-14

**Authors:** James C Moon, Daniel R Messroghli, Peter Kellman, Stefan K Piechnik, Matthew D Robson, Martin Ugander, Peter D Gatehouse, Andrew E Arai, Matthias G Friedrich, Stefan Neubauer, Jeanette Schulz-Menger, Erik B Schelbert

**Affiliations:** 1 The Heart Hospital, London, UK; 2 Institute of Cardiovascular Science, University College London, London, UK; 3 Department of Congenital Heart Disease and Pediatric Cardiology, Deutsches Herzzentrum Berlin, Berlin, Germany; 4 National Heart, Lung and Blood Institute, National Institutes of Health, Bethesda, MD, USA; 5 Oxford Centre for Clinical Magnetic Resonance Research, Division of Cardiovascular Medicine, Radcliffe Department of Medicine, University of Oxford, Oxford, UK; 6 Department of Clinical Physiology, Karolinska Institutet and Karolinska University Hospital, Stockholm, Sweden; 7 NIHR Cardiovascular BRU, Royal Brompton Hospital & Imperial College, London, UK; 8 Departments of Cardiology and Radiology, Montreal Heart Institute, Université de Montréal, Montreal, QC, Canada; 9 Department of Cardiology and Nephrology, Working Group Cardiac MRI, Humboldt University Berlin, Berlin, Germany; 10 Charite Campus Buch Experimental and Clinical Research Center, HELIOS Klinikum Berlin Buch, Berlin, Germany; 11 UPMC Cardiovascular Magnetic Resonance Center, Department of Medicine, University of Pittsburgh School of Medicine, Pittsburgh, PA, USA

## Abstract

Rapid innovations in cardiovascular magnetic resonance (CMR) now permit the routine acquisition of quantitative measures of myocardial and blood T1 which are key tissue characteristics. These capabilities introduce a new frontier in cardiology, enabling the practitioner/investigator to quantify biologically important myocardial properties that otherwise can be difficult to ascertain clinically. CMR may be able to track biologically important changes in the myocardium by: a) *native T1* that reflects myocardial disease involving the myocyte and interstitium *without* use of gadolinium based contrast agents (GBCA), or b) the *extracellular volume fraction (ECV)*–a direct GBCA-based measurement of the size of the extracellular space, reflecting interstitial disease. The latter technique attempts to dichotomize the myocardium into its cellular and interstitial components with estimates expressed as volume fractions. This document provides recommendations for clinical and research T1 and ECV measurement, based on published evidence when available and expert consensus when not. We address site preparation, scan type, scan planning and acquisition, quality control, visualisation and analysis, technical development. We also address controversies in the field. While ECV and native T1 mapping appear destined to affect clinical decision making, they lack multi-centre application and face significant challenges, which demand a community-wide approach among stakeholders. At present, ECV and native T1 mapping appear sufficiently robust for many diseases; yet more research is required before a large-scale application for clinical decision-making can be recommended.

## Who we are

The “T1 mapping development group” was informally founded October 2010. Its mission statement is “to facilitate the path of T1 mapping and ECV quantification into clinical practice”. It is aimed at academics, clinicians, pharmaceutical companies, equipment manufacturers and software developers who have a strong interest in the quantification of diffuse myocardial processes by cardiovascular magnetic resonance (CMR) with T1 mapping as their primary or core activity, and who wish to coordinate activity to the goal of being able to change therapy using these endpoints. Minutes from prior regular meetings are available [1]. The nucleus of the group and senior advisors documented in the introduction have a range of technical and clinical expertise and a broad geographical base. Together, they have provided many of the key innovations in the field. The group is now affiliated with the Society for Cardiovascular Magnetic Resonance (SCMR) and the CMR Working Group of the European Society of Cardiology.

## Background

Rapid innovations in CMR now permit the routine acquisition of quantitative measures of myocardial and blood T1 which are key tissue characteristics. T1 quantification requires the acquisition of multiple images to derive the T1 recovery curve which is governed by the exponential time constant for MR longitudinal relaxation, T1. This parameter can be displayed as a pixelwise “T1 map” whereby an estimate of T1 is encoded in the intensity of each pixel [2,3]. Its quantitative nature permits establishing normal T1 ranges, and T1 values can be assigned colors to simplify visual interpretation.

This capability introduces a new frontier in cardiology, enabling the practitioner/investigator to quantify biologically important properties of both regional and global myocardium independent of function. Historically, diffuse myocardial disease has been difficult to measure or even appreciate noninvasively. This advance is important, because focal and diffuse changes may directly reflect pathophysiologic processes across the disease spectrum from preclinical to end-stage disease. CMR may be able to track biologically important biologically important changes in the myocardium by: a) *native (noncontrast) T1* that reflects myocardial disease involving the myocyte and interstitium *without* use of gadolinium based contrast agents (GBCA), or b) the *extracellular volume fraction (ECV)* after a GBCA–a direct measurement of the size of the extracellular space, reflecting interstitial disease. The latter technique attempts to dichotomize the myocardium into its cellular and interstitial components with estimates expressed as volume fractions. The concept of employing extracellular agents to quantify the interstitial space has been exploited by investigators over many decades [4-7]. Advances in T1 measurement now permit routine noninvasive measurement of ECV.

This document provides recommendations for clinical and research T1 and ECV measurement, based on published evidence when available and expert consensus when not. We recognize *a priori* that multiple methodologies for T1 measures do and should exist, with continued evolution and residual imperfections. Furthermore, different vendor implementations of the same biomarker test may have different normal ranges. However, the rapid progress in the field renders it sufficiently mature to warrant recommendations. We make analogy to another key cardiac imaging biomarker, the left ventricular ejection fraction (LVEF), where measurement variations persist within and across modalities yet the yield of biological information is sufficient to diagnose, guide and monitor treatment and predict outcome. In fact, all imaging and non imaging biomarkers share some degree of error inherent in their measurement which is implicit in their “signal to noise” ratios and their coefficients of variation.

## Scientific and clinical relevance

### Native (Noncontrast) T1

Native (noncontrast) T1 measures of myocardium permit noninvasive detection of biologically important processes which promise to improve diagnosis, measures of disease severity, and potentially prognosis. Native T1 changes can detect pathologically important processes related to excess water in oedema [2,8,9], protein deposition [10,11], and other T1-altering substances such as lipid [12,13] or iron (hemorrhage, siderosis) [14], without the need for a GBCA. In addition native T1 techniques need not exclude patients with severe renal dysfunction. Alterations of myocardial native T1 can therefore signal both *cardiac diseases* (acute coronary syndromes, infarction, myocarditis, diffuse fibrosis causes (all high T1)) [15], and *systemic disease* such as (cardiac amyloid (high T1)) [10] Anderson-Fabry disease (low T1) [16] and siderosis (low T1). When combined in a clinical scan protocol, early evidence suggests that native T1 mapping can reveal pathology such as area at risk in acute coronary syndromes [2,8,9,17], hitherto unsuspected pathologies (global myocarditis without LGE) and preclinical disease or unsuspected cardiac involvement (iron, Fabry disease, amyloid) [10,12,18].

### ECV

The ECV technique introduces a potentially important new method to examine the myocardium because it is sensitive to the distribution of the LV myocardium into its cellular (dominated by myocyte mass) and extracellular interstitial (extracellular matrix (ECM) in the interstitium) compartments. Alterations in these compartments occur from different physiologic and pathophysiologic biologic processes [19].

Early data indicate that ECV measures appear to be as prognostically important as LVEF [20,21] which underscores the biologic importance of the interstitium. This myocyte-ECM expansion dichotomy may have important implications for identifying distinct therapeutic targets: i.e., the fibroblast versus the myocyte. This issue is especially important in heart failure where over 20 trials failed to identify therapeutic targets [22]. Furthermore, in heart failure with preserved systolic function, there are no evidence based therapies to reduce hospitalization or mortality. The extent to which primary ECM expansion from fibroblast activation drives myocyte dysfunction or the extent to which primary myocyte disease leads to ECM expansion in HF remains incompletely understood, but now the cardiology community has a developing and promising means to quantify expansion of the interstitium.

In the absence of amyloid or oedema [23], expansion of the myocardial collagen volume fraction is responsible for most ECM expansion [24] which culminates in mechanical [25-27], electrical [28-31], and vasomotor dysfunction [32], which are key elements of cardiac vulnerability [33]. ECM expansion can diminish tolerance to ischemic insults [34-36]. Other investigators have reported “vulnerable interstitium” in sudden cardiac death victims [31], and have described band-like fibrosis *in myocardium* resembling hepatic cirrhosis [37]. Thus, just like other organs, fibrosis in the myocardium is associated with cardiac dysfunction [38]. Fibrosis is associated with a number of conditions [39,40] and is considered to represent a final common pathway of myocardial disease from a variety of insults.

While late gadolinium enhancement (LGE) undoubtedly provides important diagnostic and prognostic information [41-47], T1 mapping and ECV may have an advantage over LGE for *quantifying* the degree of ECM or interstitial expansion. LGE is less suitable for quantifying extent of ECM expansion [48-54] resulting from pathologies other than myocardial infarction where the differences between normal and affected myocardium are less distinct. LGE validation data for this purpose are lacking. Spatial variation of myocardial fibrosis is the key feature that renders it potentially detectable on an LGE image [49]. In ischemic cardiomyopathy, one small study examining 10 explanted hearts reported that the majority of the total collagen content could be distributed diffusely rather than focally [39]. Such a diffuse distribution of collagen content would render its accurate quantification nearly impossible with LGE. ECV can detect early fibrosis changes not always detectable by LGE [20,49-51,55-58]. The association with outcomes appears stronger for ECV compared to LGE [20,21]. Automated parametric ECV maps are an exciting development that may facilitate rapid ECV measurement and potentially catalyze the field [59].

### Consensus terminology

To streamline the field and increase its accessibility, below are a list of recommended terms and their definitions

*Native T1 or Native myocardial T1*

- Longitudinal relaxation time (T1) values of a given tissue when no contrast agent has been applied. “Native T1” is preferred over other terms such as “pre-contrast T1” or “non-contrast T1”. If a paper is unambiguous (no contrast use, no measurement of other tissues), after initial use, native myocardial T1 can be abbreviated to simply T1.

*T1 mapping*

- A CMR method providing a parametric map whereby the T1 value is encoded in each pixel. T1 maps arise from a series of co-registered images acquired at different times of T1 recovery, typically following a magnetisation preparation by inversion or saturation. Raw images used for T1 mapping need to be acquired at identical times in the cardiac cycle. CMR methods allowing for T1 estimation from ROIs drawn in raw images of different parts of the cardiac cycle (*i.e.* not allowing pixel-based T1 analysis) should be referred to as T1 *measurements*.

*ECV or myocardial ECV*

The extracellular volume (ECV) of the myocardium reflects the volume fraction of heart tissue that is not taken by cells. This includes the intracapillary plasma volume–a significant compartment in some organs like the liver. ECV should be preferred over other terms such as “volume of distribution”. ECV does not account for regression in capillary density or other microvasculature throughout the myocardium that may be associated with adverse remodelling [60-62]. Yet, such decreases in the myocardial vasculature would only mask differences in ECM expansion that ECV attempts to measure. ECV maps can also be generated on a pixel-wise basis if native and post contrast T1 images are coregistered, quantified, and adjusted for the hematocrit [59].

*ICV or myocardial intracellular volume*

The residual of ECV (i.e., 1-ECV = ICV) representing the total tissue volume inaccessible to the GBCA molecules themselves–i.e., behind cell membranes, the composite of all cells (mostly myocyte mass, but also red blood cells, fibroblasts, macrophages, etc.). However, ICV estimates are subject to the same biases inherent in ECV estimates.

*Fibrosis*

Neither T1 mapping nor ECV directly measure the extracellular matrix or detect other important ECM qualities, such as the degree of crosslinking and post translational modification. Rather, ECV measures the space the ECM occupies which is a useful surrogate. ECV has robust histological validation as an ECM measurement which correlates with the collagen volume fraction [24,54,63]. This advance is important because myocardial fibrosis is ubiquitous and associated with myocardial remodeling [19,37,39,40,46,64,65]. In the absence of amyloidosis, other forms of infiltrative disease, or clinical conditions that would create myocardial edema, and acknowledging the other components of extracellular matrix [65], ECV is a CMR biomarker for myocardial fibrosis. We suggest avoiding use of the term “scar” due to its potential for confusion. It is not clear whether “scar” refers to: necrosis, apoptosis, or fibrosis; an ischemic etiology or non-ischemic etiology; focal or diffuse fibrosis.

### Consensus recommendations

For clinical evaluations, we recommend the following. Supporting justification for these points is provided in the subsequent section.

1. Site preparation

i. Establish site normative values for the particular set-up (vendor/field strength/magnet/contrast regime/sequence variant and patient population (age/gender)).

i. Use a validated sequence with tightly controlled protocol for the duration of the study.

i. Specify the field strength and provide a method name prominently with details about the specific pulse sequence employed to measure T1 which can affect the range of values encountered in healthy volunteers and the sensitivity to the disease process.

2. Scan types

i. For studies involving GBCA, the preferred outputs are native T1 and ECV, and not partition coefficient and post contrast T1 in isolation.

i. Haematocrit for ECV calculation should be measured contemporaneously with the CMR study.

i. A “bolus only” approach to ECV measurement is sufficient for most myocardial ECV applications.

i. For the bolus only approach, with single timepoint postcontrast measurement, a 15 minutes minimum delay should be used for ECV measures in non-infarcted myocardium [56,63,66].

i. GBCA should not be a protein bound variant for ECV measurement.

3. Scan planning and acquisition

i. Through plane partial volume averaging should be minimized by optimal slice orientation relative to the tissue (i.e., structures should be orthogonal to the imaging plane to minimize obliquity)

i. Ensure proper adjustment of shim and center frequency to minimize off resonance

i. Native and post contrast T1 maps should be acquired using the same slice prescription parameters and the same cardiac phase

4. Quality control

i. Quality measure maps such as “goodness of fit” or parameter error maps [9,18,67] should be included in the interpretation to assess the quality of acquired data. Preferably this should be performed during scanning to allow an immediate repeat of suboptimal measurements.

i. Multiple (≥2) acquisitions in different slice orientations are recommended to add diagnostic confidence.

5. Visualisation and analysis

i. T1 and ECV maps may be displayed in color (or grayscale) with appropriate scale to maximize differentiation from normal and this scale should be kept the same within a study.

i. Measurements from regions of interest should minimize in plane and through plane (obliquity) partial volume effects.

i. Regions of interest should have adequate margins of separation from tissue interfaces prone to partial volume averaging such as between myocardium and blood.

i. The exclusion/inclusion of LGE areas for ECV measures (i.e., myocardial infarction, non-ischemic LGE atypical of myocardial infarction) in T1/ECV quantification should be stated. It is acceptable for regions of interest to exclude infarcts (i.e., remote myocardium) and include non-ischaemic LGE.

6. Technical development

i. New pulse sequences and/or imaging protocols should be validated in phantoms and uniquely named.

i. T1 and T2 of phantoms should have values expected for the tissue of interest with and without contrast, at the desired field strength.

i. Evaluations should use a relevant range of heart rates. Any applied corrections, (e.g., heart rate) should be clearly defined.

i. The proposed approach should be validated against a gold standard such as spin echo relaxometry.

i. Research studies, should consider, as a guide, the general standards for the reporting of diagnostic accuracy studies (STARD) [68].

### Justification for the consensus recommendations

1. Site preparation

**Figure 1:**
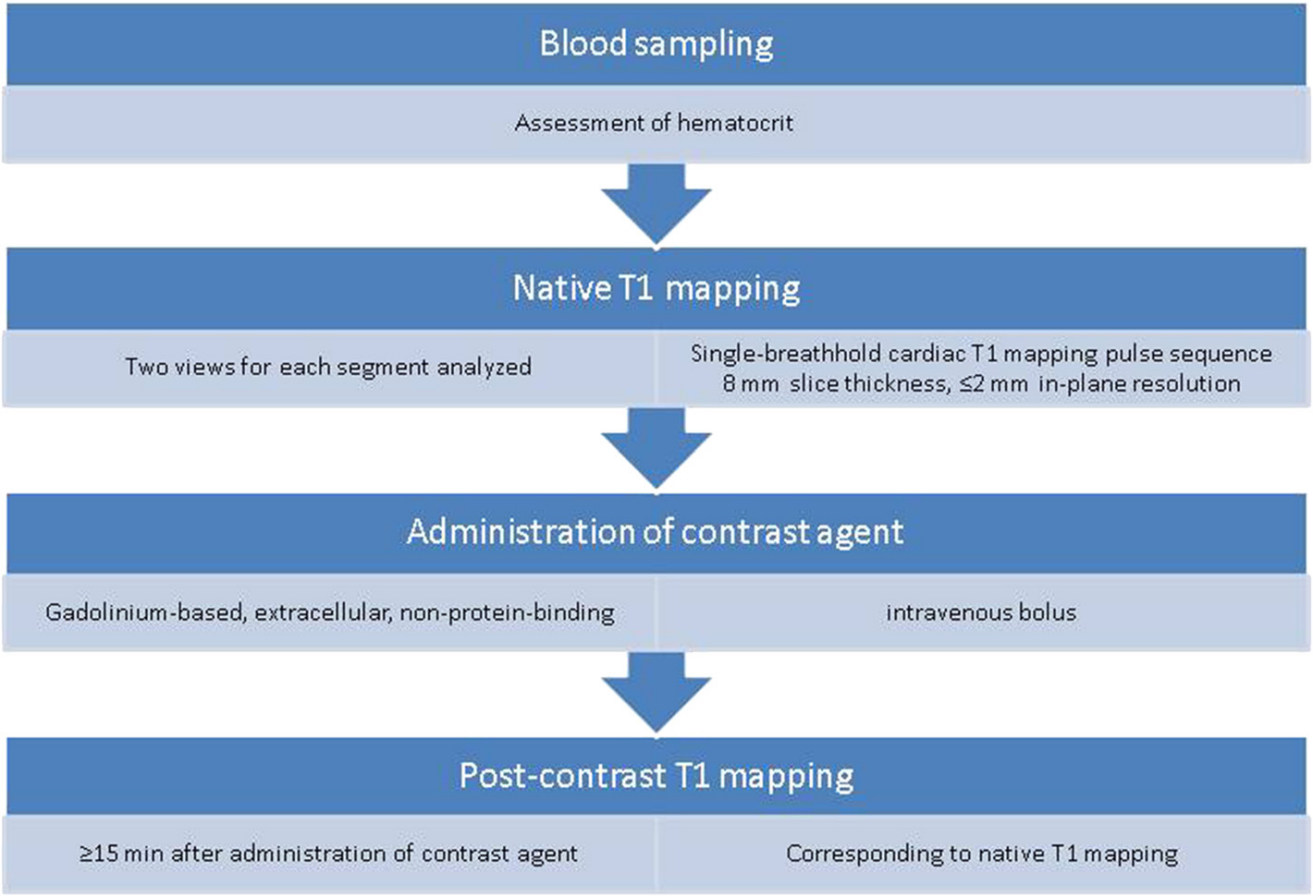
Sample scheme for measuring myocardial native T1 and ECV which can be integrated into routine CMR practice.

Significant biases in T1 measurement may depend on the specific method and imaging protocol. Caution must be exercised on relying on phantom or simulation validation which may not account for effects such as T2 relaxation or magnetization transfer (MT) which may be different in-vivo [69]. The “bias” errors may be strongly influenced by imaging parameters such as flip angle, matrix size, slice profile, and numerous other factors. The sensitivity of T1 mapping to imaging parameters is not well quantified, therefore, it is recommended to tightly control the imaging protocol for the duration of the study in order to minimize unintended variation in measured T1 due to these factors. Sequence and software upgrades, even if apparently not affecting T1 mapping (such as new tune up/shims), need to be approached with caution. Normal values for a specific protocol may vary from system to system due to changes in software versions or scanner type, will vary with field strength, and should be measured for each specific configuration. Caution is advised when sharing normative data to ensure that scanner configuration is indeed identical. Normal values should ideally be acquired on normal subject samples (with n ≥ 10, or more if small differences are being sought) representative of the target population distribution. The underlying sequence and imaging protocol should be described or referenced in sufficient detail such that it may be reproduced. A typical scan protocol is supplied, Figure 1.

2. Scan types

The relationship between the interstitium and other related parameters, the partition coefficient and isolated post contrast T1, are confounded by clinically relevant conditions arising from variation in the hematocrit, the GBCA dose which varies according to patient weight and GBCA dose conventions (0.1-0.2 mmol/kg) across centres, and the renal clearance after a bolus. Haematocrit varies considerably in large CMR cohorts [21]. To date, ECV appears better associated with patient outcomes [20,21], compared to the partition coefficient and post contrast T1. Agreement with the collagen volume fraction appears significantly higher for ECV [24,54,63] compared to isolated post contrast T1 [70,71] which does not vary linearly with GBCA concentration. Further study of these issues would be beneficial.

Equilibrium infusion does not give substantially different results to the bolus only approach except at high ECV values where an overestimation bias appears [66]. Whilst longer delays post bolus may have advantages, a 15 minute minimum appears a reasonable compromise balancing with the need for clinical throughput, and is supported by the literature [56,63,66]. Although one ECV publication [72] employed a protein bound GBCA variant, the authors pointed out that such blood protein binding renders two key assumptions invalid: firstly, the assumption of equal extracellular Gd concentrations in blood and tissue at equilibrium; secondly that the Gd relaxivity will be the same (protein binding increases relaxivity by slower molecular tumbling). Use of protein bound GBCA is not currently recommended without a more sophisticated modeling approach and more data.

3. Scan planning and acquisition

Shim. The MR reference frequency should be adjusted using a volume region over the heart both within *and through* the image slice. At higher magnetic fields shimming is particularly important to reduce non-uniformity of the resonance frequency over the heart. Through-slice extent of the volume region used can be important for two reasons: a simple frequency adjustment procedure might not be gated to the same cardiac phase, and motion in the through-plane direction can transport consequences of resonance frequency offsets [73]. Positioning the region of interest near the magnet isocenter is often beneficial [74].

Breath-hold. Failures of breath-holding will result in the later images not being registered to the initial ones, which causes substantial errors in the calculated maps. Shortened acquisition and motion correction can be beneficial but fundamentally ensuring a good breath-hold is desirable.

Timing of cardiac phase. Gating intervals (e.g., diastole) should not be changed within patients or between patients and normal controls. It is acknowledged that image field of view may need to be changed for patients. As any changes in timing parameters can potentially affect T1 measurements, modification of field-of-view are preferably done without changing acquisition matrix size or allowing any sequence timings to change.

Creation of good quality maps can only be achieved if all of the constituent images are also of good quality. The following need optimization:

4. Quality control

It is useful to establish quality measures in order to aid the interpretation and improve the confidence of T1 and ECV maps and associated measurements. In cases of suspected artefacts in T1 or ECV maps, it may be useful to examine the raw magnitude and phase images for image artefacts at the corresponding location. Parametric error maps are useful for quantitatively assessing the validity of T1 or ECV results with the direct estimate of the underlying pixel measurement precision (SD) [18,67]. The precision may be estimated from standard deviation maps generated for each T1 map [67]. Non random appearance to the error maps showing anatomy may be indicative of uncorrected motion, and thereby raise a cautionary flag in those regions. Motion correction for parametric maps, ideally employing inline automated processing as part of the pulse sequence, is desirable to minimize artefacts related to motion and misregistration, but breath-holding remains desirable. Off-resonance variation across the heart may result in regional variations in apparent T1 [74]. When available, it is useful to have field maps that may be used to ensure that apparent T1 variations are not related to systematic biases rather than true variation in T1 [74].

Although not as yet evidence based, more than one acquisition is a reasonable approach if clinical decisions are being potentially made on the quantitative results of T1 mapping. This issue is analogous to phase swaps or orthogonal cross-cuts often used for non-ischemic LGE.

5. Visualisation and analysis

During analysis, significant biases in the value of tissue T1 in a given voxel may arise due to contamination by tissue in adjacent voxels. Regions of interest (ROI) at the interface between myocardium and blood or other tissues such as fat will result in values which represent a complicated distortion of the fundamental curve-fitting assumptions and should be excluded from ROI measurements. The partial volume effect is apparent from both in-plane and through plane sources so care must be exercised to have adequate spatial resolution and slice thickness for the structures of interest, to orient slices judiciously to minimize obliquity during acquisition. During post processing, care must be exercised to draw ROIs sufficiently far from tissue interfaces which represents a key advantage of parametric maps. Criteria used for ROI delineation may have a strong influence on values and should be clearly described. These effects may not only introduce imprecision, but also bias–thinner myocardial walls (eg DCM compared to HCM, females compared to males) include proportionately more blood pool in ROIs, altering T1 (higher native, lower post contrast) and potentially inflating ECV measures.

ECV can be measured in areas of LGE; [75,76] although for ischaemic heart disease, it may reflect pathology better to dichotomise measurement into “infarct” and “remote”. For non-ischaemic cardiomyopathy, where LGE likely represents the most focal fibrosis in a continuum and where LGE extent is highly thresholding method dependent [77], LGE may be included in the ECV.

6. Technical development

Pulse sequences are continually being developed and refined, but they require initial validation prior to clinical research application. It is important that the evolution is traceable, therefore sequence naming must be unambiguous, and modifications of pulse sequences should utilize version numbers. T1 accuracy and precision should be tested systematically in sets of phantoms that should have T1 and T2 values expected for the tissue of interest with and without contrast, at the desired field strength, with temperature recordings. For example at 1.5 T, consider T1s in the range of T1 = 300-1800 ms, HR = 40-120 bpm, and with two settings of T2 (50 ms and ~180 ms). The proposed approach should be validated against a gold standard such as a series of inversion recovery spin echo acquisitions with long TR and minimal echo trains. The algorithms used for T1 estimation and any applied corrections (e.g., heart rate, systematic bias) should be fully described to allow replication and/or reversing the calculations. The STARD guidelines [68] do not specifically apply to T1 mapping, but would benefit the field as a guide, so have been included here.

### Controversies

The following areas have generated controversy and require further research.

a. What else influences what we are measuring? T1 mapping promises to improve diagnosis, improve prognosis, and inform mechanisms of disease. Robust T1 measures with high accuracy and precision would support these goals. Yet, there may be trade-offs for various T1 measuring schemes in terms of their accuracy and precision. While accuracy is desirable, precision is especially important to avoid misclassification of individuals and to stratify samples efficiently. Several factors may influence accuracy and precision of T1 measures.

The current methodology for in-vivo measurement and mapping of T1 or ECV assumes a relatively simple model that classifies a voxel as consisting of a single compartment with a homogeneous single value of the parameter T1 or ECV. According to this approximation a mono-exponential may be used to fit the measured response to either inversion or saturation recovery. In fact, we know that biological and molecular structures found in-vivo are more complex and the characteristic time constants will depend on the measurement time scales. Further, the effects such as magnetization transfer (MT), diffusion distance and time, contrast mechanisms, trans-cytolemmal water exchange rate, flow, T2 or T2* relaxation may significantly alter the apparent T1 estimates indicated by any specific method [69,78-80]. Based on these issues, there is active debate about what influences what we are really measuring, whether some methods are preferable, or whether there are still better approaches. Validation of methods based on phantoms such as agar gel is an important step but does not provide sufficient complexity to answer many of these questions. Despite this uncertainty it has been shown that current methods, if applied carefully, are reproducible [81] and are valuable tools in clinical research that correlate well with histological gold standards. In addition to the question of surrounding the simplified notion of a single T1 and how it is measured, there is also a debate over the biological influences and implications for increased or decreased T1 and/or ECV that may result with different disease states.

b. What is the best method for image acquisition?

A central question posed often to the CMR community is which method to use and more specifically which protocol to use. There are numerous approaches described in the literature and that are available to users as research “sequences” [7,51,56,80,82-85]. However, there are no current standards, and many approaches are not generally accessible. Thus, it is not possible to make a consensus recommendation on a specific method/protocol at this time. There are numerous questions/factors that are important when comparing methods such as: what influences what is really being measured, what is the accuracy and precision of the method, how reproducible is the measurement and how is it affected by variables such as motion, flow, off-resonance, how long is the measurement, and numerous others that are generally relevant in CMR. There are methods based on inversion recovery, saturation recovery, as well as hybrid methods combining inversion and saturation recovery. There are also a number of sampling and fitting strategies, and methods for image reconstruction and motion correction. Furthermore, sampling schemes could be designed differently for pre and post contrast expected T1s for optimal precision.

All of these considerations are important, and in lieu of a consensus protocol at this time, we have recommended more general guidelines for establishing normal baseline values and achieving reproducible measurements. In addition, image acquisition for T1/ECV of thin structures (RV, atria) will require new sophisticated approaches.

c. Can T1 mapping be performed in all patients?

To achieve wide clinical application, T1 mapping methods should have consistent measurement properties. Any corrections should be properly documented to assure that they can be replicated or reversed if needed. Currently, the properly breath-held single-slice 2D acquisition is preferred. This group could not identify criteria to exclude individuals given the lack of evidence. There are few data examining the degree to which respiratory motion, arrhythmia and the extremes of heart rate, perturb T1 and ECV measures. Furthermore, there are potential disease related factors–thin vs. thick myocardium; presence of arrhythmia; residual heart rate effects that could influence measured T1 and introduce bias to some unknown degree. These topics deserve further investigation

d. Which level of spatial coverage do we need?

Basal to mid short axis slices are generally preferred given their greater thickness and the generally lower obliquity compared to apical short axis slices. Thin structures and obliquity of myocardium relative to the imaging plane can introduce partial volume effects that inflate native T1 and ECV values [49,85]. Long axis slices may be more prone to errors related to through plane respiratory motion. The minimum numbers of slices and ideal slice orientation remain undefined. The solution is likely to be different for different diseases (whole heart coverage vs single representative region). These issues deserve further study. In general, for diffuse diseases the *mean* ECV or T1 seems reasonable to report while for highly regional disease, such as hypertrophic cardiomyopathy, whole heart coverage may eventually become the standard. Peak values may depend on the size of region of interest and the spatial distribution of the disease process being studied.

e. Impact of GBCA types and concentration on ECV measures?

The concentration of GBCA (or other factors such as cell size) could introduce deviations from the fast exchange limit assumption and influence how much intracellular water is relaxed in any given area of tissue due to trans-cytolemmal water exchange. “Fast water exchange” assumes water exchange is fast between intracellular and extracellular compartments relative to their differences in relaxivity [79,86]. Higher GBCA concentrations would be more prone to departure from the fast water exchange assumption and potentially measure lower ECV values [78,86]. Higher concentrations also occur post bolus with early time point measurement, renal impairment and obesity. There is some evidence for this phenomenon with upward drift of ECV measures with longer measurement times post Gd or low bolus doses [56,63,72]. Although these effects appear small, further work is needed. There are plenty of potential solutions if required (for example ECV measurement at fixed GBCA concentration; more sophisticated dosing or ECV measurement timing based on lean mass or renal function). However, none of these has been thoroughly investigated at this time.

f. How should we analyse images?

There are as yet no standard tools to analyse CMR images, with most research being performed using relatively restricted in-house developed tools, early-stage commercial packages, or manufacturer prototypes. Image analysis quality control and quantifying bias from partial volume error is not yet well developed. Automated ECV maps can speed measurement compared to ECV measures from manual regions of interest; additional advantages/disadvantages are unknown. The advantages of two timepoint (pre and post contrast) vs multi-timepoint ECV calculation are also unknown. Multi-timepoint may provide superior robustness to error estimates, but compromise potential spatial coverage and ECV map creation due to additional burden related to processing. Different situations may have different preferred approaches.. The industry is encouraged to provide highly adaptable and robust tools for standardized T1 quantification and map presentation.

g. What should we report?

The biological significance of the outputs of T1 mapping is not yet known. Multiple parameters can be reported: Global or regional ECV or T1; heterogeneity in areas with or without LGE. Currently, most studies report a singular ECV value per individual, which may be an average over short-axis slice(s) or is sometimes taken from the septum alone. A 16 segment approach however may have problems with regional measurement differences from off resonance effects or partial volume error [74].

h. Standardization for clinical utility.

Clinical delivery of T1 mapping at the level of healthcare systems to permit the change of therapy based on T1 measurements has major challenges including magnet QC, normal values and the use of multiple platforms, sequences and contrast agents. In the interim, for multicenter T1 or ECV studies, we recommend performing *stratified* statistical analyses to adjust for variation related to each site’s scanner characteristics (assuming one scanner per site). Further robust solutions will need to evolve.

i. Complementary value of multiparametric approaches: native T1, ECV, LGE and others.

Native T1 detects both intracellular and extracellular changes (focal and diffuse). ECV estimates changes in the myocardial interstitium (focal and diffuse). LGE measures focal interstitial changes. There are advantages and disadvantages to each. Native T1 has the advantage of not requiring contrast, detects iron and diffuse fat missed by the other two, but is more sensitive to changes in the type of pulse sequence, including many of its user-dependent parameters, scanner pulse sequence and T1-reconstruction implementation.

Because ECV is a ratio, ECV might be more comparable across platforms and sequences since any systematic biases in T1 estimation may cancel one another, analogous to any volumetric biases incurred during ejection fraction measures. LGE is well established and the gold standard for infarction, provides important diagnostic and prognostic information, but misses diffuse changes [41-47]. Nonetheless, ECV has advantages over LGE for *quantifying* myocardial fibrosis and the interstitial space. LGE is less suitable for quantifying lesser degrees of ECM expansion [48-54] resulting from pathologies other than myocardial infarction where the differences between normal and affected myocardium are less distinct [20,49-51,54,56,77,87,88]. ECV can detect early fibrosis changes not always detectable by LGE [20,49-51,55,56]. For every disease, the optimal use of these techniques will require exploration. These 3 techniques are also just a subset of tissue characterization techniques with other techniques (T2, diffusion, spectroscopy) and modalities (e.g., ECV by CT) [89] to be addressed in the future.

## Conclusion

Native T1 mapping and ECV may be able to provide important insights into fundamental disease processes affecting the myocardium that otherwise can be difficult to ascertain clinically. Both appear destined to affect clinical decision making but lack multi-centre application and face significant challenges, which demand a community-wide approach (MRI vendors, funding agencies, academics, software companies, contrast agent manufacturers, clinicians). At present, subject to the stated conditions, measures of ECV and native T1 mapping appear sufficiently robust for many diseases; yet more research is required before a large-scale application for clinical decision-making can be recommended. It remains the centre’s responsibility to implement quality control measures, to provide sufficient training for readers and to use validated post-processing and evaluation tools.

## Competing interests

The following interests are declared: SN, MDR, SKP: US patent pending 61/387,591: SKP: Systems and Methods for Shortened Look Locker Inversion Recovery (Sh-MOLLI) Cardiac Gated Mapping of T1. September 29, 2010. All rights sold exclusively to Siemens Medical. MDR, SKP: Patent pending 61/689,067: SKP, MDR, Color Map Design Method for Immediate Assessment of the Deviation From Established Normal Population Statistics and its Application to Cardiovascular T1 Mapping Images.

## Authors’ contributions

The nucleus members (PDG, PK, DRM, JCM, MDR, SKP, MU, EBS) convened in London, wrote sections, and edited the manuscript collated by JCM and EBS. AEA, MGF, SN, JSM, edited manuscript. The authors acknowledge reviewer input from SCMR and ESC WG during their endorsement. All authors read and approved the final manuscript.
